# Integrated stress response is critical for gemcitabine resistance in pancreatic ductal adenocarcinoma

**DOI:** 10.1038/cddis.2015.264

**Published:** 2015-10-15

**Authors:** L R Palam, J Gore, K E Craven, J L Wilson, M Korc

**Affiliations:** 1 Departments of Medicine, Biochemistry and Molecular Biology, Indiana University School of Medicine, The Melvin and Bren Simon Cancer Center and The Center for Pancreatic Cancer Research, Indianapolis, IN, USA

## Abstract

Pancreatic ductal adenocarcinoma (PDAC) is an aggressive cancer with marked chemoresistance and a 5-year survival rate of 7%. The integrated stress response (ISR) is a cytoprotective pathway initiated in response to exposure to various environmental stimuli. We used pancreatic cancer cells (PCCs) that are highly resistant to gemcitabine (Gem) and an orthotopic mouse model to investigate the role of the ISR in Gem chemoresistance. Gem induced eIF2 phosphorylation and downstream transcription factors ATF4 and CHOP in PCCs, and these effects occurred in an eIF2*α*-S51 phosphorylation-dependent manner as determined using PANC-1 cells, and wild type and S51 mutant mouse embryo fibroblasts. Blocking the ISR pathway in PCCs with the ISR inhibitor ISRIB or siRNA-mediated depletion of ATF4 resulted in enhanced Gem-mediated apoptosis. Polyribosomal profiling revealed that Gem caused repression of global translation and this effect was reversed by ISRIB or by expressing GADD34 to facilitate eIF2 dephosphorylation. Moreover, Gem promoted preferential mRNA translation as determined in a TK-ATF4 5′UTR-Luciferase reporter assay, and this effect was also reversed by ISRIB. RNA-seq analysis revealed that Gem upregulated eIF2 and Nrf2 pathways, and that ISRIB significantly inhibited these pathways. Gem also induced the expression of the antiapoptotic factors Nupr1, BEX2, and Bcl2a1, whereas ISRIB reduced their expression. In an orthotopic tumor model using PANC-1 cells, ISRIB facilitated Gem-mediated increases in PARP cleavage, which occurred in conjunction with decreased tumor size. These findings indicate that Gem chemoresistance is enhanced by activating multiple ISR-dependent pathways, including eIF2, Nrf2, Nupr1, BEX2, and Bcl2A1. It is suggested that targeting the ISR pathway may be an efficient mechanism for enhancing therapeutic responsiveness to Gem in PDAC.

Pancreatic ductal adenocarcinoma (PDAC) is characterized by marked chemoresistance, an overall 5-year survival rate of 7%, and a median survival of 6–7 months.^1^ Gemcitabine (Gem) is often the standard of care for patients with locally advanced or metastatic PDAC. Gem and nab-paclitaxel combine to improve median survival to 8.5 *versus* 6.7 months with Gem alone.^2^

The integrated stress response (ISR) is a signaling pathway initiated upon phosphorylation of the alpha subunit of eukaryotic initiation factor 2 (eIF2*α*) at serine 51 in response to diverse stress conditions. There are four mammalian eIF2 kinases that phosphorylate eIF2*α*: general control non-derepressible 2 (GCN2), which is upregulated by amino acid starvation; protein kinase R (PKR), which is activated by viral infections; PKR-like endoplasmic reticulum (ER) kinase (PERK), which is upregulated by ER stress; and heme-regulated eIF2*α* kinase (HRI), which is induced upon oxidative stress or heme deprivation.^3, 4, 5, 6^ Phosphorylated eIF2*α* (p-eIF2*α*) markedly attenuates translation initiation and overall protein synthesis, allowing for conservation of cellular resources. Moreover, p-eIF2*α* facilitates the preferential translation of certain mRNAs, most notably ATF4, whose upregulation promotes the expression of genes involved in oxidative stress (OS), metabolism, and nutrient uptake.^7, 8, 9^ Thus, gene reprograming by p-eIF2*α* allows cells to recover from stress-induced damage facilitating survival in response to mild stress and promoting apoptosis in response to chronic stress.^9, 10^

PDAC is associated with constitutive activation of several pro-survival pathways including AKT, NFkB, and STAT3.^11, 12^ PDACs also harbor major driver mutations, including *Kras* (~95%) and *p53* (~75%), which may also contribute to apoptosis resistance.^13, 14^ Pancreatic cancer cells (PCCs) also exhibit enhanced autophagy, and their chemoresistance may be enhanced by autophagy.^15, 16^ However, suppressing autophagy for therapeutic purposes may be associated with enhanced tumor growth and chemoresistance.^17^

The ISR pathway is activated in response to various cellular stresses such as hypoxia and nutrient deprivation,^18, 19^ conditions that exist within the PDAC microenvironment. Accordingly, in the present study, we conducted *in vitro* and *in vivo* studies and RNA-seq analysis to explore the possibility that Gem activates an antiapoptotic response in PCCs via the ISR pathway. We now report that ISRIB enhances Gem chemosensitivity in PCCs by suppressing ISR and its downstream antiapoptotic pathways.

## Results

### Gemcitabine activates the integrated stress response pathway in pancreatic cancer cells

**Figure 1:**
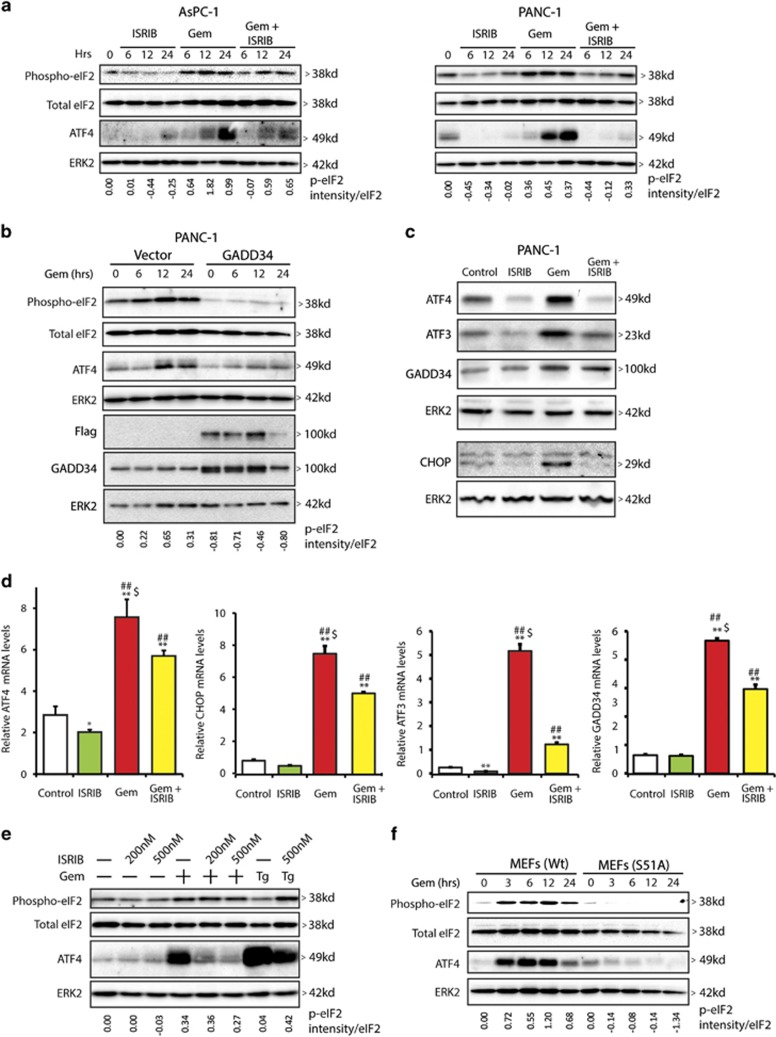
Gemcitabine induces the integrated stress pathway in pancreatic cancer cells. (**a**) AsPC-1 and PANC-1 cells were incubated with 0.5 *μ*M ISRIB or 10 *μ*M Gem for indicated times and protein lysates were analyzed by immunoblotting for phospho-eIF2, total eIF2, and ATF4 using specific antibodies. ERK2 served as loading control. (**b**) Empty vector or GADD34 expression plasmid DNA was transfected into PANC-1 cells, which were incubated 24 h later with 10 *μ*M Gem. Protein lysates were analyzed by immunoblotting. (**c**) PANC-1 cells were incubated for 36 h with 0.5 *μ*M ISRIB or 10 *μ*M Gem and protein lysates were analyzed by immunoblotting for ATF4, ATF3, GADD34, and CHOP using specific antibodies. ERK2 served as loading control. (**d**) PANC-1 cells were incubated with 0.5 *μ*M ISRIB or 10 *μ*M Gem for 24 h. ATF4, CHOP, ATF3, and GADD34 mRNA levels were measured using qRT-PCR. Relative actin mRNA levels were used for normalization. Data are the means±S.D. from three experiments. *P<0.05, **P<0.001 compared with control, ^##^*P*<0.01 compared with ISRIB, and ^$^*P*<0.05 compared with Gem+ISRIB. (**e**) MEFs were incubated for 6 h with 10 *μ*M Gem in the absence and presence of ISRIB. As a positive control, MEFs were also incubated for 6 h with thapsigargin (0.1 *μ*M). (**f**) Wild-type MEFs or MEFs mutated for eIF2*α* at serine 51 (to alanine) were incubated with 10 *μ*M Gem for indicated time point and analyzed by immnuoblotting. Panels (**a**, **b**, **d** and **e**) show representative data from three independent experiments

Gem induces oxidative stress in PCCs,^20, 21^ and oxidative stress can activate ISR pathways.^8^ It is not known, however, whether Gem's actions in PCCs are modulated by ISR signals. We therefore studied the effects of ISRIB, a novel ISR inhibitor,^22^ on Gem-activated stress pathways. In ASPC-1 and PANC-1 human PCCs, eIF2 phosphorylation was decreased at 6, 12, and 24 h following ISRIB addition (Figure 1a). By contrast, Gem increased p-eIF2 and ATF4 protein levels at the same time points (Figure 1a), whereas ISRIB attenuated these Gem-induced effects (Figure 1a), indicating that ISRIB attenuates p-eIF2 and downstream events in PCCs. Given that GADD34 delivers protein phosphatase-1 (PP1) to the vicinity of eIF2 and facilitates PP1-mediated p-eIF2 dephosphorylation,^23^ it was of interest to determine whether eIF2 dephosphorylation was similarly modulated by GADD34 in PCCs. Indeed, when PANC-1 cells were transfected with a GADD34 cDNA, there was a marked reduction in eIF2 phosphorylation which was associated with marked decreases in ATF4 protein levels (Figure 1b).

We next sought to assess the effects of Gem and ISRIB on additional stress pathway modulators. By comparison to control PANC-1 cells, Gem induced 4-fold and 10-fold increases in ATF4 and ATF3 mRNA levels, and 8-fold and 6-fold increases in CHOP and GADD34 mRNA levels, respectively (Figure 1d). Gem also increased the levels of all four proteins (Figure 1c). Although ISRIB slightly decreased basal ATF4 and ATF3 mRNA and protein levels, it did not alter CHOP or GADD34 mRNA levels in PANC-1 cells, but it significantly attenuated Gem's ability to upregulate these mRNAs and proteins (Figure 1c).

ISRIB suppresses signaling downstream of p-eIF2 but is not known to alter either basal or stress-induced eIF2 phosphorylation.^22^ In view of ISRIB's ability to decrease both basal and Gem-induced p-eIF2 in PCCs (Figure 1a), we sought to determine whether ISRIB also modulates eIF2 dephosphorylation in mouse embryo fibroblasts (MEFs). ISRIB did not alter basal or Gem-induced eIF2 phosphorylation in MEFs (Figure 1e), but markedly attenuated Gem-induced increases in ATF4 protein levels (Figure 1e). Thapsigargin (0.1 *μ*M), an ER stress-inducer, caused a robust increase in ATF4 levels compared with Gem, suggesting that Gem-induced stress is relatively mild with respect to the ISR pathway. To confirm that p-eIF2 modulates ATF4 expression in MEFs, we examined Gem actions in MEFs that express wild-type eIF2*α* (MEF-S/S) or a mutated eIF2*α* in which serine 51 was mutated to alanine (MEF-A/A). Gem readily induced eIF2 phosphorylation and upregulated ATF4 protein levels in MEF-S/S cells, but not in MEF-A/A cells, confirming that Gem-mediated upregulation of ATF4 is dependent on phospho-eIF2 (Figure 1f).

### ISRIB enhances the inhibitory actions of gemcitabine on pancreatic cancer cell survival

**Figure 2:**
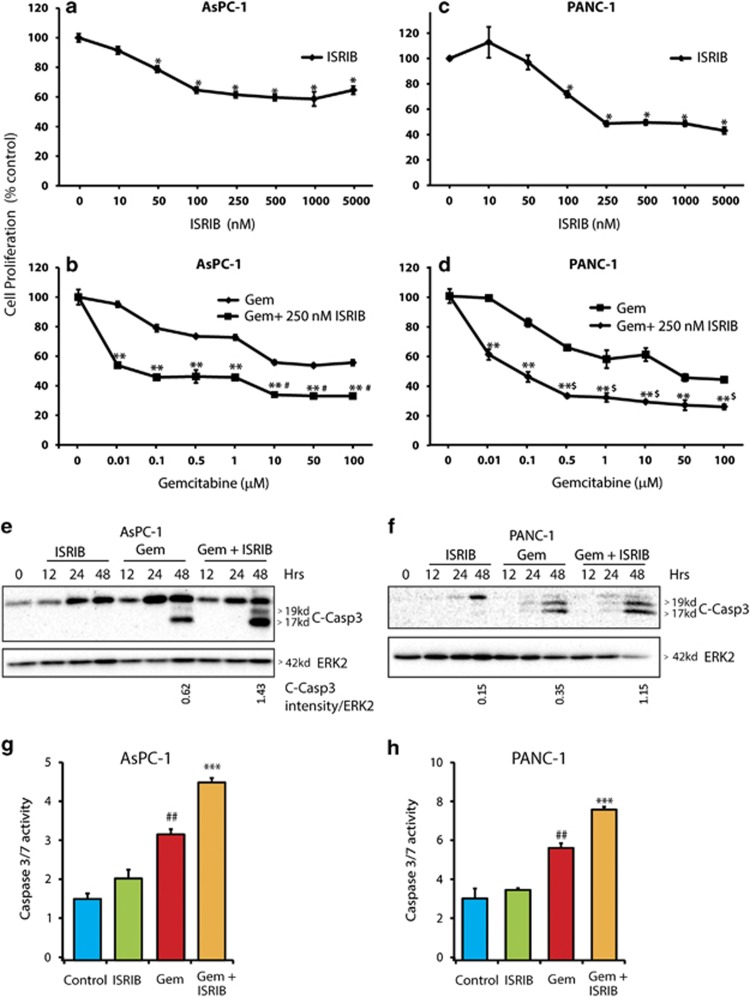
Suppression of integrated stress pathway enhances gemcitabine chemosensitivity. (**a**) AsPC-1 cells were incubated with ISRIB alone or (**b**) Gem alone, or ISRIB and Gem for 72 h as indicated, and MTT assays were performed. Data are the means±S.D. from three experiments. *P<0.001 compared with control, **P<0.005 compared with Gem alone, ^#^*P*<0.005 compared with Gem (1 *μ*M)+ISRIB (250 nM). (**c**) PANC-1 cells were incubated with ISRIB alone or (**d**) with Gem alone or with Gem+ISRIB for 72 h as indicated, and MTT assays were performed. Data are the means±S.D. from three experiments. *P<0.001 compared with control, **P<0.005 compared with Gem alone, ^$^*P*<0.005 compared with 0.1 *μ*M Gem+250 nM ISRIB. (**e** and **g**) AsPC-1 cells were incubated with 0.5 *μ*M ISRIB alone or in combination with 10 *μ*M Gem. (**f** and **h**) PANC-1 cells were incubated with 0.5 *μ*M ISRIB alone or in combination with 1 *μ*M Gem. Cell lysates were immunoblotted for cleaved caspase-3 protein in (**e** and **f**) and caspase-3/7 activity was measured in (**g** and **h**). Data shown are from three independent experiments in (**e** and **f**). Data are the means±S.D. from three experiments in ( **g** and **h**). ^##^*P*<0.01 compared with control and ISRIB, ***P<0.01 compared with control, ISRIB, and Gem

To determine whether inhibition of ISR pathways in PCCs modulates their survival, cells were incubated for 72 h with 10 nM to 5 *μ*M ISRIB. The effective concentration of ISRIB required for inhibition of AsPC-1 and PANC-1 cell survival was 250 nM (Figures 2a and c). Next, both cell lines were incubated for 72 h with 0.01 *μ*M to 100 *μ*M Gem, in the absence or presence of 250 nM ISRIB for 72 h. Both AsPC-1 and PANC-1 cells were relatively resistant to Gem's inhibitory actions, exhibiting IC-50 values of 50 and 15 *μ*M, respectively (Figures 2b and d). In combination with 250 nM ISRIB, the half-maximal inhibitory concentration of Gem in both cell lines decreased to 100 nM (Figures 2b and d). Moreover, in the presence of ISRIB, Gem's maximally effective growth inhibitory concentration was 10 and 0.5 *μ*M in ASPC-1 and PANC-1 cells, respectively (Figures 2b and d). To assess whether the decrease in cell survival was due to enhanced apoptosis, cleaved caspase-3 levels and caspase activity were examined (Figures 2e–h). Although ISRIB alone did not induce caspase-3 cleavage or caspase-3/7 activity in either cell line, it increased Gem's effects on cleaved caspase-3 levels and significantly enhanced caspase activity in both cell lines when compared to Gem alone (Figures 2e–h).

### Gemcitabine represses global mRNA translation while preferentially enhancing ATF4 translation

**Figure 3:**
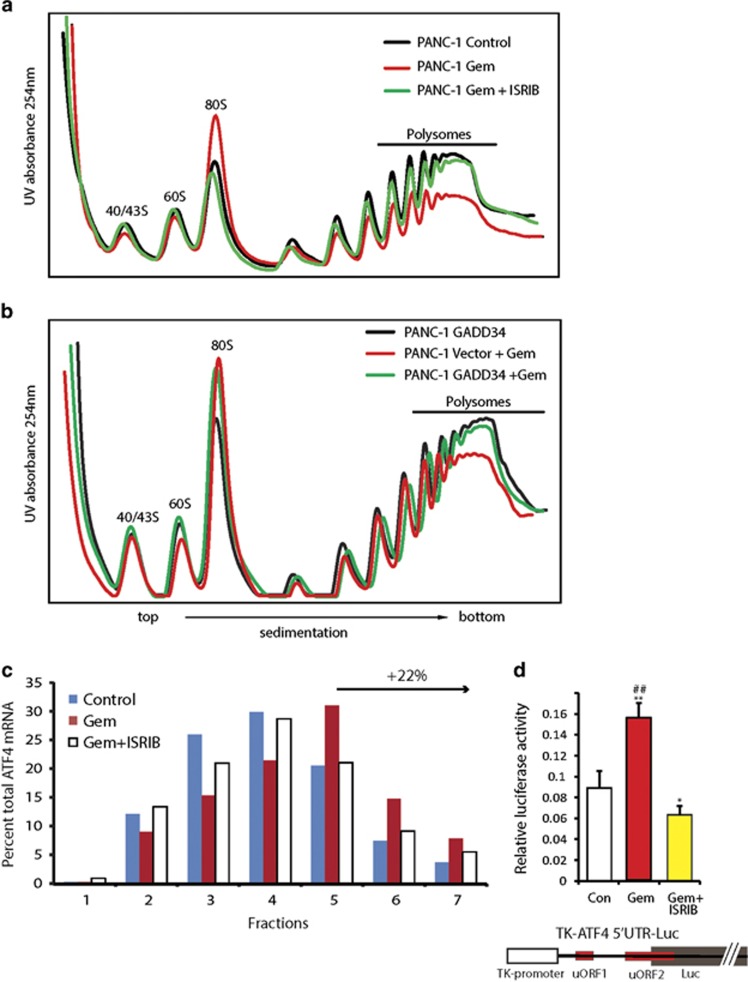
Gemcitabine causes translation repression in pancreatic cancer cells. (**a**) PANC-1 cells were incubated for 12 h in the absence or presence of 10 *μ*M Gem or 0.5 *μ*M ISRIB. Cell lysates were subjected to sucrose gradient centrifugation, and gradients were fractionated in-line with 254 nm UV absorbance measurement. Top fractions containing free ribosomal subunits are labeled as 40/43S, 60S. Mono ribosomes are labeled as 80S. Ribosomes bound to RNA fractions were labeled as polysomes. (**b**) PANC-1 cells were transfected with an empty vector or the GADD34 plasmid DNA, and incubated 24 h later with 10 *μ*M Gem for 12 h. Cell lysates were analyzed for polysome profiles as in (**a**). (**c**) PANC-1 cells were incubated in the absence or presence of Gem or ISRIB+Gem and subjected to sucrose gradient centrifugation as in (**a**). Fractions were collected and luciferase (10 ng/ml) spiked into each fraction. RNA Isolated and ATF4 transcript levels were quantitated using qRT-PCR, and normalized to spike-in luciferase control. The percent total ATF4 transcript for each fraction is represented. Fractions 5, 6, and 7 corresponds to fractions with polysomes rich in translation. (**d**) PANC-1 cells were transfected with the TK-ATF4-Luc plasmid construct that with illustrated features: ATF4 5′UTR harboring uORF1 and uORF2, TK promoter and Luciferase coding region. At 24 h post transfection cells were incubated for 12 h in the absence or presence of 10 *μ*M Gem and 0.5 *μ*M ISRIB. Firefly luciferase units were measured and normalized to internal control Renilla luciferase activity. Data are the means ±S.D. of three experiments. *P<0.05, **P<0.005 compared with control, ^##^*P*<0.001 compared with Gem+ISRIB. Panels **a** and **b** show representative data from three independent experiments

Stress-induced eIF2 phosphorylation enhances ATF4 and CHOP expression at the mRNA and protein levels, but reduces global protein synthesis, thereby conserving cellular energy.^3, 24^ ISRIB markedly reduced Gem-induced ATF4 protein levels, but only slightly decreased Gem-induced ATF4 mRNA upregulation (Figure 1). Given the inability of ATF4 mRNA to be translated into protein in the presence of ISRIB, it is likely that ISRIB reprograms this pathway at a translational level. To assess this possibility, polyribosomal profiling was performed, revealing that Gem did not alter the sedimentation profiles of 40/43S and 60S ribosomal subunits, but increased the monosome 80S peak, pointing to a Gem-induced translation initiation defect (Figure 3a). Moreover, Gem markedly decreased the number of polyribosome-associated mRNAs when compared with control cells, whereas ISRIB completely reversed these Gem-induced alterations (Figure 3a). Thus, Gem suppresses global protein synthesis and ISRIB reverses this block.

GADD34 is upregulated by Gem and this effect is partially reversed by ISRIB (Figure 1c). Therefore, we sought to assess the consequences of transient overexpression of GADD34 on Gem-induced changes in polyribosomal sedimentation. GADD34 expression did not alter the sedimentation profile of PANC-1 cells and did not affect Gem-induced increases in the monosome 80S peak (Figure 3b). By contrast, GADD34 mimicked the effect of ISRIB and reversed the Gem-induced decrease in mRNA-associated polyribosomes (Figure 3b). Thus, GADD34 attenuated translation repression in response to Gem, confirming that eIF2 phosphorylation is essential for this Gem-induced action. Similar results were observed with Gem in wild-type MEFs but not in mutated MEFs (MEF-A/A) (Supplementary Figure S1). ATF4 preferentially associates with polyribosomes during stress-induced eIF2 phosphorylation.^25^ To confirm this, we measured ATF4 mRNA distribution across polyribosome fractions using sucrose density centrifugation. By comparison to control, polysome-associated ATF4 transcript levels were enhanced by 22% in Gem-treated PANC-1 cells, but this increase did not occur when ISRIB was combined with Gem (Figure 3c). Furthermore, Gem enhanced the readout of a TK-ATF4 5′UTR-Luciferase reporter assay,^25^ and this effect was completely reversed by ISRIB (Figure 3d), confirming that Gem-induced stress also acted to promote preferential translation of mRNAs.

### Gemcitabine and ISRIB interact to modulate multiple signaling cascades

**Figure 4:**
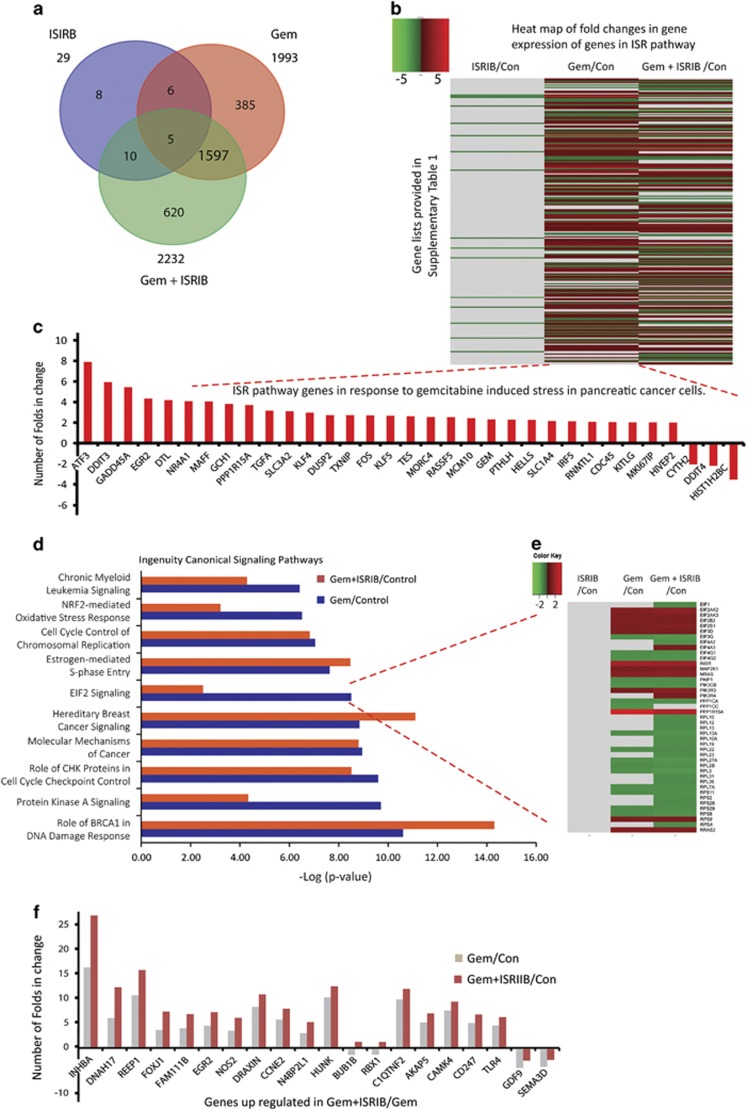
Genome-wide analysis for response to gemcitabine and ISRIB. PNAC-1 cells were incubated for 36 h in the absence or presence of 10 *μ*M Gem 0.5 *μ*M ISRIB, and collected RNA was used for RNA transcriptome sequencing. (**a**) Total number of genes with significant change in gene expression (>1.5-folds increase or <−1.5-fold decrease, *P*<0.001) following indicated incubations compared with control are represented in a Venn diagram. (**b**) Heat map for fold changes in gene expression (*P*<0.001) over control following indicated incubations is shown for genes involved in the ISR pathway. ISR genes and gene expression data from RNA-seq analysis are shown. (**c**) ISR genes with more than 2-fold change in gene expression (*P*<0.001) in response to Gem compared with control are shown. (**d**) IPA was performed on obtained RNA-seq data and major canonical signaling pathways that were modulated in response to drugs are represented. (**e**) Heat map for fold changes in gene expression (*P*<0.001) over control condition following indicated incubations is shown for genes involved in eIF2 signaling. Network of genes involved in eIF2 signaling was collected from IPA as described in Materials and Methods. (**f**) Top 20 genes that are differentially regulated in response to Gem alone *versus* Gem+ISIRB are shown (*P*<0.001)

To assess the actions of Gem and ISRIB on gene expression, RNA-Seq data from PANC-1 cells incubated with Gem and/or ISRIB were analyzed next. Gem and ISRIB altered the expression of 1993 and 29 genes (>1.5-fold increase or <−1.5-fold decrease, *P*<0.001), respectively, compared with control (Figure 4a). Out of 1993 genes altered by Gem, 690 were downregulated and 1303 were upregulated, and from the ISRIB-responsive genes, 27 were downregulated and 2 were upregulated. By contrast, the combination of Gem and ISRIB altered the expression of 2232 genes (1425 genes up, 807 genes down, *P*<0.001), and 620 of these genes (~28%) were modulated by the combination of Gem and ISRIB (Figure 4a). Genes modulated downstream of eIF2 signaling were identified from a previous study that used a genetically engineered artificial eIF2 kinase in MEFs that express wild type or mutated eIF2*α*.^9^ Expression analysis following Gem-induced ISR revealed that 225 ISR-associated genes^9^ were significantly modulated (*P*<0.001; Figure 4b, Supplementary Table 1). This list included such genes as ATF3, CHOP (DDIT3), GADD34 (PPP1R15A), GADD45a, SLC3A2, TXNIP, and SLC1A4 (Figures 4b and c), and ISRIB antagonized these Gem-induced changes (Figure 4b). Using quantitative RT-PCR (qRT-PCR) we validated the RNA-seq data by measuring multiple transcripts downstream of p-eIF2, including ATF3, CHOP, GADD34, and other well-known ATF4-regulated genes such as ASNS, TRIB3, GAS-5, SLC7A11, SESN2, STC2, and SAT1. qRT-PCR confirmed that Gem enhanced the levels of all of these mRNAs, and that ISRIB attenuated these effects (Supplementary Figure S4a). Moreover, Gene Set Enrichment Analysis (GSEA) of p-eIF2-dependent genes^9^ within our RNA-Seq data set from ISRIB, Gem, and Gem+ISRIB treated cells revealed negative (NES: −3.63, *P*-value <0.001), positive (NES: 2.88, *P*-value <0.001), and negative (NES: −2.14, *P*-value <0.005) enrichment of eIF2 signaling, respectively, suggesting that ISRIB can attenuate Gem-induced eIF2 signaling (Supplementary Figure S4b).

Ingenuity Pathway Analysis (IPA) confirmed that multiple pathways were modulated by Gem, and that ISRIB enhanced Gem-mediated expression of genes implicated in BRCA1's DNA damage response and hereditary breast cancer signaling (Figure 4d). However, ISRIB markedly attenuated Gem's effects on the expression of genes involved in eIF2 signaling, NRF2-mediated oxidative stress responses, and protein kinase A pathways (Figure 4d). The most dramatic inhibitory changes occurred with genes involved in eIF2 signaling (Figures 4d and e, Supplementary Table 2). Thus, ISRIB suppressed Gem-induced expression of ribosome-related genes such as 60S ribosomal protein L10 (RPL10) and ~12 related RPL members, 40S ribosomal protein S11 (RPS11) and four related RPS members, as well as the expression of genes implicated in signaling,^26^ such as those encoding phosphatidylinositol-4,5-bis-phosphate-3-kinase, catalytic subunit beta (PK3CB), and serine/threonine-protein phosphatase PP1-alpha catalytic subunit (PPP1CA). Some genes were upregulated by Gem and Gem+ISRIB, but were not previously known to be ISR related. These include inhibin A which may contribute to cancer-associated cachexia,^27^ nitric oxide synthase-2 (NOS2) which may enhance resistance to apoptosis,^28^ CCNE which encodes cyclin E1, and Nedd4-binding protein 2-like 1 (N4BP2L1) which is a Bcl3-binding protein (Figure 4f) whose upregulation may lead to increased sensitivity to platinum.^29, 30^

### Gemcitabine induces antiapoptotic Nupr1 and pro-survival factors via the ISR pathway

**Figure 5:**
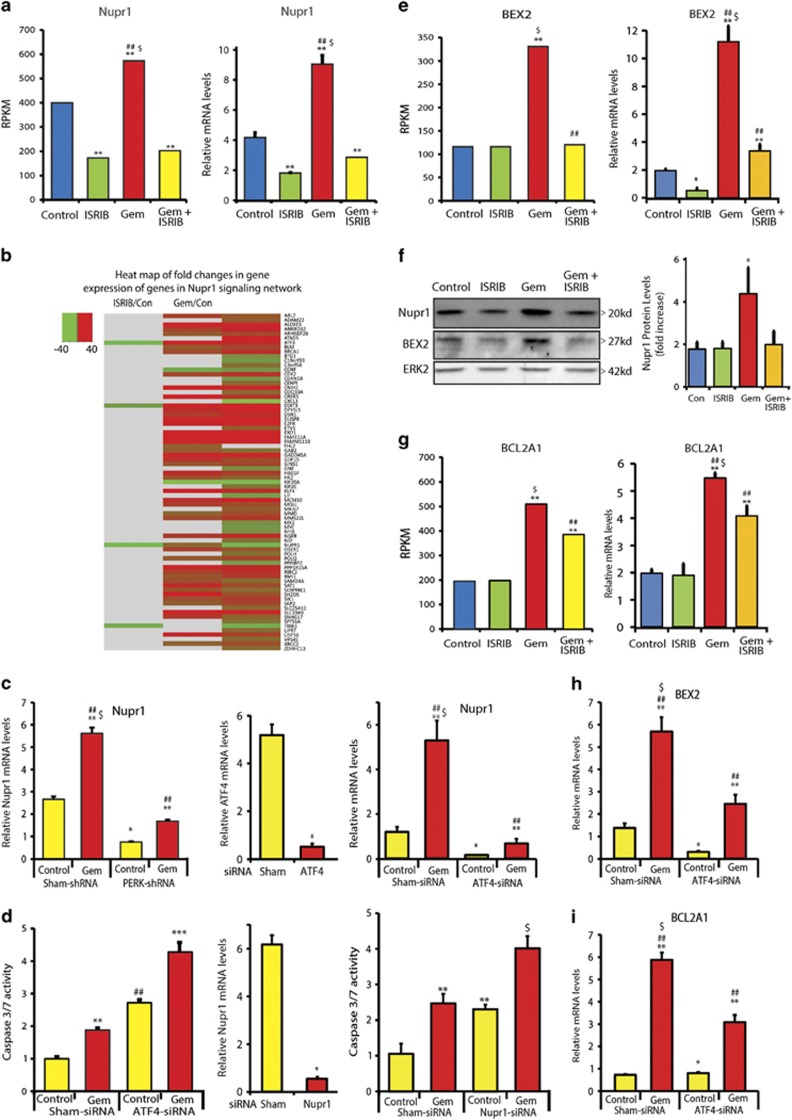
Inhibition of ISR pathway completely reduces gemcitabine-induced Nupr1 increase in PANC-1 cells. (**a**) Nupr1 expression data from RNA-seq analysis and qRT-PCR is represented. **P<0.001 compared with control, ^##^*P*<0.001 compared with ISRIB, ^$^*P*<0.001 compared with Gem+ISRIB. PANC-1 cells were incubated with 0.5 *μ*M ISRIB, 10 *μ*M Gem, or ISRIB+Gem for 36 h, and RNA was analyzed for Nupr1 mRNA levels using qRT-PCR. Data are the means ±S.D. of three experiments. **P<0.001 compared with control, ^##^*P*<0.001 compared with ISRIB, ^$^*P*<0.001 compared with Gem+ISRIB. (**b**) Heat map for fold changes in gene expression in response to indicated drugs over control for genes involved in Nupr1 network, based on Nupr1 network genes collected from Ingenuity Pathway Analysis as described in Materials and Methods. (**c**) PANC-1 cells stably expressing either control-shRNA or PERK-shRNA and PANC-1 cells with ATF4 siRNA were incubated with 10 *μ*M Gem for 24 h, and RNA was analyzed for Nupr1 mRNA expression levels. Data are the means ±S.D. of three experiments. **P<0.001 compared with control-shRNA and PERK-shRNA, ^##^*P*<0.001 compared with PERK-shRNA, *P<0.001 compared with control-shRNA, ^$^*P*<0.001 compared with PERK-shRNA in the presence of Gem. (**d**) PANC-1 cells were transfected with indicated siRNA, and incubated 48 h later in the absence or presence of 10 *μ*M Gem for 24 h. Caspase-3 activity was measured using Casp3/7 glow assay. Data are the means ±S.D. from three independent experiments. **P<0.001 compared with control, ^##^*P*<0.001 compared with sham-siRNA control and sham-siRNA with Gem, ***P<0.001 compared with sham-siRNA control, ATF4 siRNA control, and sham-siRNA with Gem, ^$^*P*<0.001 compared with sham-siRNA control, Nupr1-siRNA control, and sham-siRNA with Gem. (**e**) Antiapoptotic factor BEX2 and (**g**) BCL2A1 expression data from RNA-seq analysis and qRT-PCR analysis are shown; **P<0.001 compared with control and ISRIB, ^##^*P*<0.001 compared with Gem, ^$^*P*<0.001 compared with Gem+ISRIB. (**f**) PANC-1 cells were incubated with 0.5 *μ*M ISRIB or 10 *μ*M Gem for 36 h and protein lysates were analyzed by immunoblotting for Nupr1 and BEX2 using specific antibody. ERK2 served as loading control. Nupr1 protein levels were quantified from the blots and fold changes in expression are shown. Data are the means±S.D. of three experiments. *P<0.01 compared with control, ISRIB, and Gem+ISRIB. (**h** and **i**) PANC-1 cells expressing ATF4 siRNA were incubated with 10 *μ*M Gem for 24 h, and RNA was analyzed for (**h**) BEX2 mRNA and (**i**) BCL2A1 expression levels. Data are the means ±S.D. of three experiments. **P<0.001 compared with control siRNA and ATF4 siRNA, ^##^*P*<0.001 compared with ATF4 siRNA, *P<0.001 compared with control siRNA, ^$^*P*<0.001 compared with ATF4 in the presence of Gem

Nuclear protein 1 (Nupr1), also known as p8, is a helix–loop–helix protein that is overexpressed in PDAC and that enhances pancreatic tumorigenesis by suppressing oncogenic K-ras-induced senescence and inhibiting apoptosis.^31^ Nupr1 also promotes Gem chemoresistance in PCCs by inhibiting stress-induced cell apoptosis,^32, 33^ whereas ATF4/CHOP signaling induces Nupr1 expression in response to amino acid deprivation.^34^ Our RNA-seq data revealed that ISRIB suppressed both basal and Gem-stimulated Nupr1 mRNA levels, and qRT-PCR and immunoblotting confirmed these results (Figures 5a and f). IPA of RNA-seq data confirmed that Gem induced alterations in the expression of Nupr1 network genes and that ISRIB blocked this effect (Figure 5b, Supplementary Table 4). We therefore sought to determine whether Nupr1 contributes to the ISR's role in Gem chemoresistance, by first assessing whether Nupr1 expression is modulated by PERK or ATF4. PERK knockdown, which reduced Gem-induced CHOP upregulation (Supplementary Figure S2), and ATF4 knockdown both suppressed basal and Gem-stimulated Nupr1 levels (Figure 5c). Moreover, ATF4 or Nupr1 knockdown both enhanced Gem-stimulated caspase-3/7 activity (Figure 5d). Together, these findings demonstrate that Nupr1 enhances chemoresistance downstream of the ISR.

Gem also enhanced the expression of the antiapoptotic factors brain expressed X-linked 2 (BEX2) and Bcl-2 related protein A1 (BCL2A1), which was completely blocked by ISRIB (Figures 5e–g), and markedly suppressed by ATF4 knockdown (Figures 5h and i). Thus, the ISR upregulates these genes, in part, through ATF4. In addition, Gem increased the expression of genes involved in reactive oxygen species (ROS) reduction, such as cystathionine gamma lyase (CTH), solute carrier family 7 member 11 or xCT (SLC7A11), and sestrin2 (SESN2), and genes related to glucose deprivation and nutrient stress such as phosphoenol carboxykinase2 (PCK2), branched chain amino acid transaminase 1 (BCAT1), phosphoglycerate dehydrogenase (PHGDH), and phosphoserine aminotransferase 1 (PSAT1) (Supplementary Figure S3). qRT-PCR validated the RNA-seq data, confirming that ISRIB completely blocked upregulation of these transcripts (Supplementary Figure S4). Thus, by upregulating ATF4, Gem activates apoptosis resistance, and oxidative and nutrient stress response pathways that are attenuated by ISRIB.

### Inhibition of gemcitabine induced ISR pathway attenuates tumor growth *in vivo*

**Figure 6:**
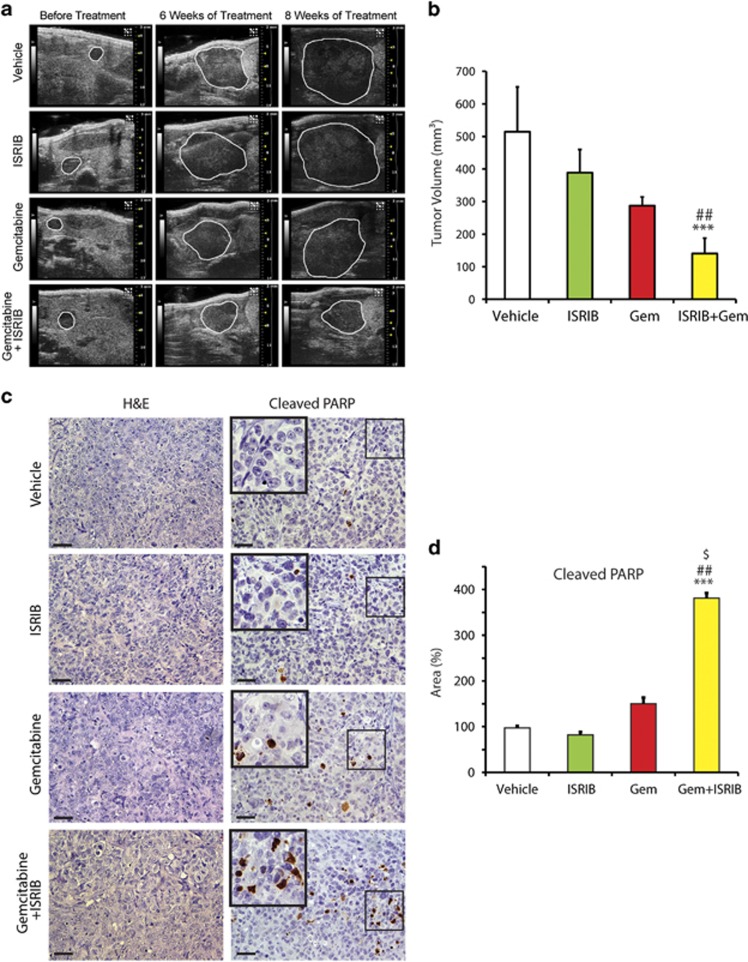
Inhibition of gemcitabine induced ISR pathway decreases PANC-1 orthotopic tumor growth. (**a**) PCCs were injected into the pancreas of athymic mice as described in Materials and Methods. High-resolution ultrasound images were obtained at 2 weeks post-PCC injection, and at indicated treatment times. Tumors are outlined and are representative images from six mice per group. (**b**) Tumor volumes were calculated using 3-D abdominal imaging. There was a step-wise decrease in tumor volumes from Vehicle-treated, to ISRIB-treated, to Gem-treated, and to Gem+ISRIB-treated mice. Data are the means±S.D.; ***P<0.05 compared with control, ^##^*P*<0.05 compared with ISRIB. (**c**) H&E and PARP staining was performed on paraffin-embedded tissue sections (0.5 *μm* thick), bars=50 *μ*m. (**d**) PARP-positive cells were counted and tabulated after normalization to percent of control. Data are the means±S.D. for four experiments. ***P<0.001, ^##^*P*<0.001, ^$^*P*<0.001 compared with control, ISRIB, and Gem+ISRIB treatments, respectively

We next examined PANC-1 cell-derived tumor growth in an orthotopic nude mouse model. All the animals had approximately the same tumor size prior to treatment initiation (Figure 6a). ISRIB alone or Gem alone did not significantly reduce tumor volumes by comparison with the control group (Figure 6a). By contrast, concomitant treatment with Gem and ISRIB significantly reduced tumor volumes after 8 weeks, when compared with control, Gem, or ISRIB treatment groups (Figures 6a and b). Although Gem, ISRIB, or their combination did not alter tumor (Figure 6c) or adjacent normal pancreas histology (Supplementary Figure S5), there was a marked increase in the number cells that were positive for cleaved PARP in mice treated with Gem and ISRIB, when compared with control, Gem, or ISRIB treated groups (Figures 6c and d). By contrast, the number of phospho-histone H3-positive cells was similar in all four groups (Supplementary Figure S5). Thus, combined Gem and ISRIB therapy enhances apoptosis *in vivo* without altering proliferation.

## Discussion

Cancers exhibit self-sufficiency, unlimited cell growth, sustained ability to obtain nutrients, apoptosis resistance, capacity to invade and metastasize, and insensitivity to growth inhibitory pathways.^35^ In PDAC, these aberrant processes are associated with multiple and diverse genetic alterations impacting ~12 major signaling pathways.^36, 37, 38^ Despite numerous clinical trials, current therapies for metastatic PDAC are having limited success. Thus, Gem prolongs life by 6–8 weeks, while the addition of erlotinib or nab-paclitaxel adds 12 days or 15 weeks, respectively, to overall survival.^39^ In addition, the combination of 5-fluorouracil (5-FU), leucovorin, irinotecan, and oxaliplatin (FOLFIRINOX), which is generally restricted to patients with excellent performance status, increases survival by 18 weeks.^40^ Although patients with a BRCA mutation signature may respond to either platinum drugs or PARP inhibitors,^38^ there are no curative therapies in PDAC, underscoring the urgent need for innovative therapeutic strategies.

The etiology for therapeutic failure in PDAC is multifactorial. The abundance of gene mutations and overexpression of receptors and ligands results in the activation of aberrant signaling pathways that enhance the resistance of PCCs to chemotherapeutic agents,^35, 36, 37, 38^ whereas the desmoplastic and hypoxic tumor microenvironment impedes efficient drug delivery into the pancreatic tumor mass.^41, 42^ In the case of Gem, resistance may be due to various mechanisms, such as decreased expression of equilibrative nucleoside transporter-1 (hENT1), decreased deoxycytidine kinase (dCK) activity which translates into attenuated dCK activation of Gem, and increased ribonucleotide reductase (RR) expression which leads to more efficient DNA repair due to increased generation of deoxyribonucleoside triphosphates.^13, 43^ Moreover, Gem-induced upregulation of DUSP1 results in activation of a negative feedback loop that promotes chemoresistance,^44^ upregulation of HMGA1 enhances pro-survival signals through an Akt-dependent mechanism,^45^ and increased expression of multidrug resistance (MDR) genes including ABCC1, ABCC3, ABCC5, and ABCB1 promotes drug efflux.^46^ MUC4 promotes PDAC growth and metastasis, and negatively regulates equilibrative/concentrative nucleoside transporter (hCNT1) levels in PCCs via NF-*κ*B, thereby conferring Gem resistance.^47^ MUC4 also attenuates mitochondrial cytochrome *c* release and caspase-9 activation by inactivating the pro-apoptotic protein Bad.^48^ Gem also induces ROS which upregulate CXCR4 levels in PCCs via mechanisms involving activation of NF-*κ*B.^49^

In the current study we uncovered a novel mechanism whereby Gem activated a cytoprotective ISR pathway in PCCs by inducing eIF2 phosphorylation and downstream ISR targets. We also demonstrated that blocking ISR pathway activation with the symmetric bisglycolamide ISRIB greatly enhanced Gem sensitivity by promoting PCC apoptosis *in vitro* and *in vivo*. Moreover RNA-seq data on ISRIB-treated PANC-1 cells showed reduced levels of genes involved in amino acid and glucose metabolism such as PHGDH, PCK2, aldehyde dehydrogenase 1 family member2 (ALDH1L2), asparagine synthase (ASNS), PSAT1, and SLC1A4 (a glutamate/neutral amino acid transporter) (Supplementary Table 5). ISIRB also inhibited expression of genes that regulate protein synthesis such as ribosomal protein S6 kinase A2 (RPS6A2) (Supplementary Figure S3). It is therefore possible that downregulation of these key genes contributes to ISRIB-mediated inhibition of PCC proliferation *in vitro*.

ISRIB also reversed Gem-induced translation repression, but the molecular mechanism underlying this effect is not known. Nonetheless, recent reports suggest that ISRIB action intervenes at the level of eIF2-phosphorylation-mediated stress granule formation, thereby reversing stress-induced translation repression.^50^ Moreover, stress granule formation transiently protects cytosolic RNAs from harmful effects during cell stress.^51^ Therefore, it is possible that the observed translation repression in PCCs may be due to Gem-induced p-eIF2mediated stress granule formation. ISIRB also promotes eIF2 guanine nucleotide exchange factor (eIF2GEF) activity towards eIF2, thereby inhibiting the ISR pathway.^52^ It is therefore possible that ISRIB may also act in a similar manner in PCCs. While further studies are necessary to explore these possibilities, ISRIB alone modulated the expression of 29 genes (*P*<0.001), but when combined with Gem, ISRIB altered expression of a much larger set of genes (Figure 4a), including genes not previously reported to be associated with ISR pathways (Figure 4f, Supplementary Table 3). Thus, ISRIB interferes with PCC adaptation to stress.

The chromatin factor Nupr1 is essential for survival of PCCs exposed to stress, and high Nupr1 levels are associated with poor prognosis in PDAC.^32, 33^ Moreover, Nupr1 deficiency sensitizes PCCs to Gem and to other novel nucleoside analogs, such as Ly101-4b and WJQ63.^32^ In the present study we decreased Nupr1 levels by targeted siRNA, or by inhibiting the ISR pathway with ISRIB or ATF4-targeting siRNA; each approach sensitized PCCs to Gem. These observations are concordant with earlier studies using MEFs and NIH3T3 cells, which showed that nutrient deprivation triggers Nupr1 expression via ATF4/CHOP, and that the Nupr1 promoter has the CHOP binding site ATTGCATCA.^34^ Moreover, our RNA-seq data and qRT-PCR validation demonstrated that Gem increases Nupr1 levels via the ISR pathway. Taken together with our PERK and ATF4 knockdown studies, these findings suggest that the PERK>eIF2-P>ATF4>CHOP pathway is involved in Nupr1 transcriptional regulation in PCCs.

In addition to enhancing cell survival through the above mechanisms, Gem upregulated the antiapoptotic proteins BEX2 and Bcl2A1, and these actions were suppressed by ISRIB or ATF4 knockdown. BEX2 inhibits mitochondrial apoptosis in breast tumors and plays a role in G1 cell cycle progression.^53, 54^ BEX2 also upregulates the pro-survival factor Bcl-2 and downregulates pro-apoptotic factors BAD, BAK1, and PUMA.^53^ Similarly, Bcl2A1 is an antiapoptotic protein that is highly expressed in many cancers.^55^ It has been implicated in tumorigenesis and chemoresistance,^55^ as well as resistance to the Bcl-2/Bcl-Xl inhibitor ABT-737.^56^ In the present study, ISRIB was well-tolerated in an orthotopic tumor model. There was increased PARP cleavage in the tumors without decreased proliferation in response to combined Gem/ISRIB treatment compared to either drug alone, suggesting that reduced tumor growth was due to enhanced apoptosis. Previous studies have demonstrated that the PERK specific inhibitor GSK2656157 reduces cancer cell growth *in vivo* by impairing angiogenesis and reducing amino acid metabolism.^57^ However, administration of GSK2656157 to mice for 2 weeks caused pancreatic degeneration and other long-term toxicities.^57, 58^ By contrast, ISIRB is well-tolerated and restores protein synthesis without causing pancreas toxicity.^59^ Our study is in agreement with these observations since ISRIB did not impact the normal pancreatic parenchyma adjacent to the orthotopic tumors. Taken together, our findings indicate that Gem-induced ISR pathway activation promotes chemoresistance by upregulating multiple antiapoptotic factors such as Nupr1, Bcl2A1, BEX2, and NRF2, and that ISR pathway inhibition with ISRIB or similar molecules could be a novel mechanism for enhancing Gem chemosensitivity in PDAC, and could greatly increase the therapeutic effectiveness of Gem-based combinatorial therapies.

## Materials and Methods

### Ethics statement

All animal experiments conducted at Indiana University were approved by the Institutional Animal Care and Use Committee of Indiana University.

### Cell culture

AsPC-1 and PANC-1 human PCCs were obtained from the American Type Culture Collection (Manassas, VA, USA). Mouse embryo fibroblasts (MEFs) were a kind gift from Dr. Ronald Wek (IU School of Medicine, Indianapolis, IN, USA).^60^ PANC-1 and MEF cells were cultured in DMEM, and AsPC-1 cells were cultured in RPMI 1640. Media were supplemented with 5% (PCCs) or 10% (MEFs) FBS, 100 units/ml penicillin and 100 *μ*g/ml streptomycin. Cells were incubated at 37 °C in a humidified 5% CO_2_ incubator.

### Transfection and transduction

PANC-1 cells were GFP labeled using a lentiviral vector GFP plasmid DNA (Open Biosystems, Pittsburgh, PA, USA). shRNA expression plasmids (TRCN0000262379; Sigma, St Louis, MO, USA) were used to silence PERK eIF2 kinase by lentiviral transduction.^61^ The GADD34 DNA fragment which was amplified from PANC-1-derived cDNA was inserted into pcDNA4 at Not1 and XHO1 sites. An N-terminal flag tag was added using phospho primers, and the start codon context in GADD34 coding sequence was changed to a strong Kozak context (GCCACCAUGG) to achieve high GADD34 expression after sequence confirmation (Sangers method; IU DNA sequencing core). siRNA specific for ATF4 (SR300328c), non-targeting control siRNA (Origene, Rockville, MD, USA), or siRNA specific for Nupr1 (SI02664333; Qiagen, Valencia, CA, USA) was transfected (20 nM each) into PANC-1 cells at 70% confluence using lipofectamine-2000 (Life technologies, Grans Island, NY, USA).

### Immunoblotting

Immunoblotting was performed^62^ using antibodies against: ATF4 (11815), phospho-eIF2 (3398), total eIF2 (9722), and Casp3 (9661) all from Cell Signaling Technology (Danvers, MA, USA); Flag-M2 (F1804) from Sigma-Aldrich (St Louis, MO, USA); Nupr1 (ab6028) from Abcam (Cambridge, MA, USA); Bex2 (sc48966), ATF3 (sc188), GADD34 (sc825), CHOP (sc7351), and ERK2 (sc154) were all from Santa Cruz Biotechnology (Dallas, TX, USA).

### Statistical analysis

SigmaPlot (Systat Software Inc., San Jose, CA, USA) was used for one-way ANOVA. *P-*values are shown in Figures 5e, 6b and d, Supplementary Figure S4. Student's *t*-test was used for data in Figures 1c, 3, 5b and c, Supplementary Figure S2. For RNA-seq data, the DESeq algorithm^63^ were used, providing *P*-values for differential gene expression as shown in respective figures. We used six mice per group for *in vivo* studies based on a calculation of 80% power to detect any tumor size difference greater than 18% with a 5% type I error rate, based on tumor size variability (9.8%) in PANC1 tumors.

### 3-(4,5-Dimethylthiazol-2-yl)-2,5-diphenyltetrazolium bromide (MTT) and Casp3/7 glow assays

MTT assays were performed as described.^62^ Caspase cleavage was measured using the caspase-3/7 glow assay (Promega, Madison, WI, USA).

### Luciferase reporter assay

TK-ATF4-5′UTR-Luc plasmid was a kind gift from Dr. Ronald Wek. TK-ATF4-Luc was transfected into PANC-1 cells (80% confluent) using Lipofectamine-2000. Lysates were assayed for luciferase activity using Dual-Luciferase assay system (Promega) and a Synergy H1 hybrid reader (Biotek, Winooski, VT, USA).

### RNA preparation and transcriptome data analysis (RNA-seq)

PANC-1 cells were incubated for 36 h in the absence or presence of 10 *μ*M Gem and/or 0.5 *μ*M ISRIB. RNA was collected using Trizol (Catalog # 15596-018, Invitrogen, Grand Island, NY, USA), treated with DNAse using DNase Kit (Catalog # AM1907, Ambion, Grand Island, NY, USA) and re-purified using RNeasy kit (Catalog # 74106, Qiagen). Total RNA (5 *μ*g) was used for transcriptome analysis. Read count quantification of triplicate RNA samples from each treatment condition was performed using the Illumina HiSeq 2000 platform (BGI-Americas, Cambridge, MA, USA). Differential expression analysis was performed using the DESeq algorithm.^63^ ISR pathway network gene information (Figure 4b) was obtained as follows: 654 probe sets from the mouse Affymetrix U74Av2 chip respective to eIF2-phosphorylation-dependent genes were downloaded,^9^ and the Affymetrix NetAffx tool was used to map these probesets to their latest gene mappings (641 genes). NCBI's file for *Mus musculus* was used to find the associated Mouse Genome Informatics (MGI) ids, yielding 636 associated MGI ids. The HMD Human Phenotype.rpt file from the MGI Data and Statistical Reports site served to map the 636 MGI ids to 586 unique human genes. Eight genes without RNA-Seq information and 346 genes that were not differentially expressed were also removed from the list. The remaining 232 genes were differentially expressed (*P*<0.0001) in at least one of the three comparisons. An eIF2 network of 68 gene symbols (Figure 4e, Supplementary Table 2), and a Nupr1 network of 205 gene symbols (Figure 5d, Supplementary Table 4) were obtained from IPA and the associated Entrez gene ids were queried using NCBI's Homo sapiens gene information. Of the 68 eIF2 network genes, 43 were differentially expressed (*P*<0.0001) in at least one of the three comparisons. In the Nupr1 gene network, three genes no longer had associated Entrez gene ids, and two were not differentially expressed. The remaining 200 genes were differentially expressed (*P*<0.0001) in at least one of the three comparisons. For all three networks, the fold change values as compared with the untreated condition were plotted in Figures 4b and e, and 5d, as a gradient from green (downregulated) to red (upregulated) if *P*<0.001, and gray otherwise, using heatmap.2 in the R gplots package.^64^ For GSEA of RNA-Seq data custom gene set was prepared with top 500 genes from eIF2- phosphorylation-dependent genes that were downloaded.^9^ Using the GSEA pre-ranked tool, GSEA version 2.1.0 was run on ranked lists of genes, sorted according to their modified log2 change differences, from the following RNA-seq comparisons: ISRIB *versus* Control, Gem *versus* Control, Gem *versus* Gem+ISRIB.

### Reverse transcription and real-time qPCR

RNA was isolated using Qiagen-RNAeasy isolation kit (Qiagen), and quantified using Nanodrop (Wilmington, DE, USA). RNA (2 *μ*g) was used for cDNA preparation using RNA to cDNA reverse transcription kit (Life Technology, Grand Island, NY, USA). 2x power SYBR Green enzyme mix or Fast enzyme mix and Taqman primers (Applied Biosystem, Grand Island, NY, USA) were used for quantitation of mRNA levels by real-time PCR. PCR cycles and signal acquisition were performed using Vii-Applied Biosystem thermal cycler (Applied Biosystem). Primers used for quantitation of mRNA levels ATF4: F-ccaacaacagcaaggaggat, B-ggggcaaagagatcacaagt; CHOP: F-agaaccaggaaacggaaacaga, B-tctccttcatgcgctgcttt; Gas5: F-cctcaaacttctgggctcaa, B-tcaggcagtctacaaagaccac; Asns: F-tacaaccacaaggcgctaca, B-aagggcctgactccataggt; Trib3: F-gtcttcgctgaccgtgaga, B-cagtcagcacgcaggactc; Slc7a11: F-gggcatctctctgaccatct, B-tcccaattcagcataagacaaa; Sesn2: F-tccgccactcagagaagg, B-ggagggcgtacagcagag; Stc2: F-atattgtacagtcctttcgaccatt, B-cgtgcgtgtgtatgagtgtg; Sat1: F-gaggttccttgggtcatgg, B-gtggttcctcattcgtctcc; BEX2: F-gatgcagaaaatggtggtttg, B-cctctttggactccattactcc; Bcl2a1: F-caggagaatggataaggcaaa, B-accagcataggtgtgtgattgt; ACTB: F-gagcgcggctacagctt, B-tccttaatgtcacgcacgattt. Taqman primers used were as follows; GADD34: Hs00169585_m1, ATF3: Hs00231069, Nupr1: Hs01044304, ACTB: Hs01060665_g1.

### Polysome profile preparation

Polysome profile was prepared as described previously.^60^ Briefly, PANC-1 cells were cultured as described above and treated with 10 *μ*M Gem or 0.5 *μ*M ISRIB or in combination for 12 h. For the experiment involving GADD34 expression, PANC-1 cells were transfected with GADD34 expression plasmid, and 24 h post transfection the cells were incubated in the presence or absence of 10 *μ*M Gem for 12 h. Cycloheximide (10 *μ*g/ml) was added 10 min prior to cell harvesting. Cells were then washed with cold phosphate-buffered saline (pH 7.4) solution containing 10 *μ*g/ml cycloheximide and cell lysates were prepared in a buffer solution containing 20 mM Tris–HCl (pH 7.5), 5 mM MgCl_2_, 100 mM NaCl, and 0.4% Nonidet P-40 supplemented with 50 *μ*g/ml cycloheximide. Cell lysates were passed through a 23-gauge needle, incubated on ice for 10 min, pre-cleared by a 10- min centrifugation (10 000 r.p.m., 4 °C), and layered onto a 10–50% sucrose gradient solution containing 20 mM Tris–HCl (pH 7.5), 5 mM MgCl_2_, 100 mM NaCl, and 10 *μ*g/ml cycloheximide. Sucrose gradients were then subjected to a 2- h centrifugation in a Beckman SW-41Ti rotor (40 000 rpm, 4 °C). Gradients were fractionated using Biocomp Gradient Station (Fredericton, NB, Canada) and absorbance of RNA at 254 nm was recorded using an in-line UV monitor. Synthetic poly (A)+ luciferase RNA (Promega) was spiked (10 ng/ml) into each collected fractions, and RNA was isolated from these fractions using Trizol-LS reagent (Invitrogen). Single-stranded cDNA prepared using Superscript III reverse transcriptase (Invitrogen). ATF4 mRNA transcript levels were quantified using qRT-PCR, and data normalized with the spike-in luciferase mRNA levels. Percent of total ATF4 mRNA in each fraction is represented in the figure.

### Orthotopic implantation of tumor cells and treatment schedule

PANC-1 cells (0.5X10^6^) expressing a Lenti-GFP plasmid were suspended in 0.1 ml sterile PBS, and injected into the pancreas of 8-week-old nude mice (Harlan, Indianapolis, IN, USA) as reported.^65^ Tumors were imaged 15 days later, using the Vevo2100 high-resolution ultrasound (V-US) (Visual Sonics Inc., Toronto, Canada). Tumor volumes were calculated based on acquired 3-D abdominal scans. Mice were divided into four groups (6 mice/group). On day 18 post-surgery, twice-weekly intraperitoneal drug administration was initiated. Group 1 received Gem (Biotang, Waltham, MA, USA), at a dose of 50 mg/kg body weight dissolved in saline^44^ and DMSO containing PEG-400 (Sigma). Group 2 received 2.5 mg/kg body weight ISRIB (Xcessbio, San Diego, CA, USA) dissolved in DMSO containing PEG-400 and saline.^22^ Group 3 received both Gem and ISRIB, whereas Group 4 received saline and DMSO containing PEG-400. Tumor volumes were determined by V-US at 8 weeks post-surgery. Mice were then killed and tumor tissues were collected for analysis.

### Immunohistochemistry

Orthotopic tumors were fixed in 10% formalin and embedded in paraffin. H&E staining and immunohistochemistry were performed using 5 *μ*m-thick sections,^65^ anti-phospho-histone H3 (Novacastra Leica Microsystems, Buffalo Grove, IL, USA) and anti-cleaved PARP (Asp175; Cell Signaling Technology, Danvers, MA, USA) antibodies. Sections were incubated in HRP-labeled secondary antibody and staining was detected by DAB (Dako, Carpinteria, CA, USA). Images were taken using an Olympus BX60 microscope (Olympus, Center Valley, PA, USA) equipped with a QImaging EXI Blue camera and ImagePro software (Media Cybernetics, Atlanta, GA, USA).

## Abbreviations

ATF3: activating transcription factor 3
ATF4: activating transcription factor 4
Bcl2A1: Bcl-2 related protein A1
BEX2: brain expressed X-linked 2
CHOP: C/EBP homologous protein
eIF2: eukaryotic initiation factor 2
ER: endoplasmic reticulum
GADD34: growth arrest and DNA damage-inducible protein 34
GCN2: general control non-derepressible 2
Gem: Gemcitabine
ISR: integrated stress response
ISRIB: integrated stress response inhibitor
Nrf2: nuclear Factor, erythroid 2-like 2
Nupr1: nuclear protein, transcriptional regulator 1
PCCs: pancreatic cancer cells
PDAC: pancreatic ductal adenocarcinoma
PERK: PKR-like endoplasmic reticulum kinase
RNA-seq: RNA sequencing
SLC7A11: solute carrier family 7 member 11
siRNA: small interfering RNA
TK: thymidine kinase promoter
