# Supplementary material for: Integrated stress response is critical for gemcitabine resistance in pancreatic ductal adenocarcinoma
Source: Cell Death Dis. 2015 Oct 15;6(10):e1913–. doi: 10.1038/cddis.2015.264 (PMC4632294; doi:10.1038/cddis.2015.264)
Supplement: Supplementary Legends [file cddis2015264x1.docx]

**­­­­Supplementary legend:**

Supplementary Figure S1: Gem causes translation repression in mouse embryo fibroblasts. a) MEFs were incubated for 6 hours in the absence or presence of 10 µM Gem. Cell lysates were subjected to sucrose gradient centrifugation, and gradients were fractionated in-line with 254nm UV absorbance measurement. Top fractions containing free ribosomal subunits are labeled as 40/43s, 60s. Mono ribosomes are labeled as 80s. Ribosomes bound to RNA fractions labeled as polysomes. b) MEFs harboring mutated eIF2 at serine 51 were incubated for 6 hours in the absence or presence of 10 µM Gem. Polysomal profiles were prepared as described in panel a. Panel a and panel b show representative data from three independent experiments.

Supplementary Figure S2: Inhibition of PERK pathway reduces Gem-induced CHOP increase in PANC-1 cells. PANC-1 cells stably expressing either control-shRNA or PERK-shRNA were prepared as described in Methods. Cells were then incubated with 10 μM Gem for 24 hours and derived RNA was analyzed for PERK and CHOP expression by qRT-PCR. Data are the means ±SD from 3 independent experiments. **p<0.001 compared to control-shRNA and PERK-shRNA, ^##^p<0.001 compared to PERK-sh RNA, *p<0.001 compared to control-shRNA, $p<0.001 compared to PERK-shRNA and Gem.

Supplementary Figure S3: ISRIB reduces Gem-induced expression of pro-survival genes. PANC-1 cells were incubated with 0.5 µM ISRIB or 10 µM Gem, or ISRIB + Gem for 36 hours. Derived RNA was subjected to RNA-seq analysis as described in Methods. Differential expression analysis between the groups was performed using the DESeq algorithm as mentioned in methods. Normalized RPKM values for indicated mRNA transcript are shown. **p<0.001 compared control, ^##^p<0.001 compared ISRIB, $p<0.001 compared to Gem + ISRIB.

Supplementary Figure S4: ISRIB reduces Gem-induced expression of eIF2 phosphorylation dependent genes. a) RNA-seq data was validated using qRT-PCR method. PANC-1 cells were incubated with 0.5 µM ISRIB or 10 µM Gem, or ISRIB + Gem for 36 hours. RNA isolated, and analyzed for indicated transcripts using qRT-PCR method. Actin was used as normalization control. Data are the means ±SD from 3 independent experiments. **p<0.01 compared to control, ^##^p<0.01 compared to ISRIB, ^$^p<0.001 compared to Gem + ISRIB. b) ISRIB attenuates Gem-induced eIF2 Signaling in PANC-1 cells. (i) ISRIB treated PANC-1 cells are negatively enriched in an eIF2 signaling signature. Genes known to be upregulated by eIF2 signaling are down-regulated by ISRIB treatment when compared to control. (NES: -3.63, FWER p-Value < 0.001). (ii) Gem treated PANC-1 cells are positively enriched in an EIF2 signaling signature. Genes up-regulated by EIF2 signaling are also up-regulated by Gem treatment when compared to control. (NES: 2.88, FWER p-Value < 0.001). (iii) Gem + ISRIB treated PANC-1 cells are negatively enriched in an eIF2 signaling signature. Genes known to be up-regulated by eIF2 signaling are down-regulated by Gem + ISRIB treatment when compared to Gem-treated cells. (NES: -2.14, FWER p-Value < 0.005)

Supplementary Figure S5: ISRIB does not alter normal pancreas histology and does not suppress proliferation *in vivo*. a) Tumor histology. H&E and phospho histone H3 staining was performed on paraffin embedded tissue sections (0.5 µM thick), Bars=50 μm. b) Phospho histone H3 positive cells were quantified and normalized to percent of control. Data are the means ± SE for 4 experiments. C) H&E staining was performed on paraffin embedded orthotopic tumor tissue sections (0.5 µM thick), Bars = 50 μm. Low magnification images (left panels) show that there are regions of normal pancreas adjacent to the orthotopic tumors (outlined). High magnification images (right panels) show that in these regions, islets (dashed outline), ducts (arrowheads) and the surrounding acini are normal, and appear similar when comparing control-treated mice with, ISRIB-, Gem- and Gem + ISRIB-treated mice.

Supplementary Table1 (XLS file): ISR pathway genes involved in Gem response. List of ISR pathway genes shown in Figure 4b Heat map is presented. Gene expression is expressed as log ratio of fold changes compared to control.

Supplementary Table2 (XLS file): eIF2 related genes involved in Gem response. List of genes in eIF2 related gene network from IPA analysis as shown in Fig4e Heat map is presented. Gene expression is shown as log ratio of fold changes compared to control.

Supplementary Table3 (XLS file): Differentially expressed genes in PCCs in response to Gem and ISRIB. List of differentially expressed genes in Gem + ISRIB combination vs Gem in PANC1 cells is shown. Gene expression is shown as fold changes compared to control.

Supplementary Table 4 (XLS file): Nupr1 related genes involved in Gem response. List of genes in Nupr1 related gene network from IPA analysis as shown in Fig5d Heat map. Gene expression is shown as log ratio of fold changes compared to control.

Supplementary Table 5 (XLS file): Differentially expressed genes in response to ISRIB only treatment. List of genes differentially expressed in response to ISRIB in PANC1 cells is presented. Gene expression is shown as log ratio of fold changes compared to control.
