# Supplementary figures and images for: Integrated stress response is critical for gemcitabine resistance in pancreatic ductal adenocarcinoma
Source: Cell Death Dis. 2015 Oct 15;6(10):e1913–. doi: 10.1038/cddis.2015.264 (PMC4632294; doi:10.1038/cddis.2015.264)

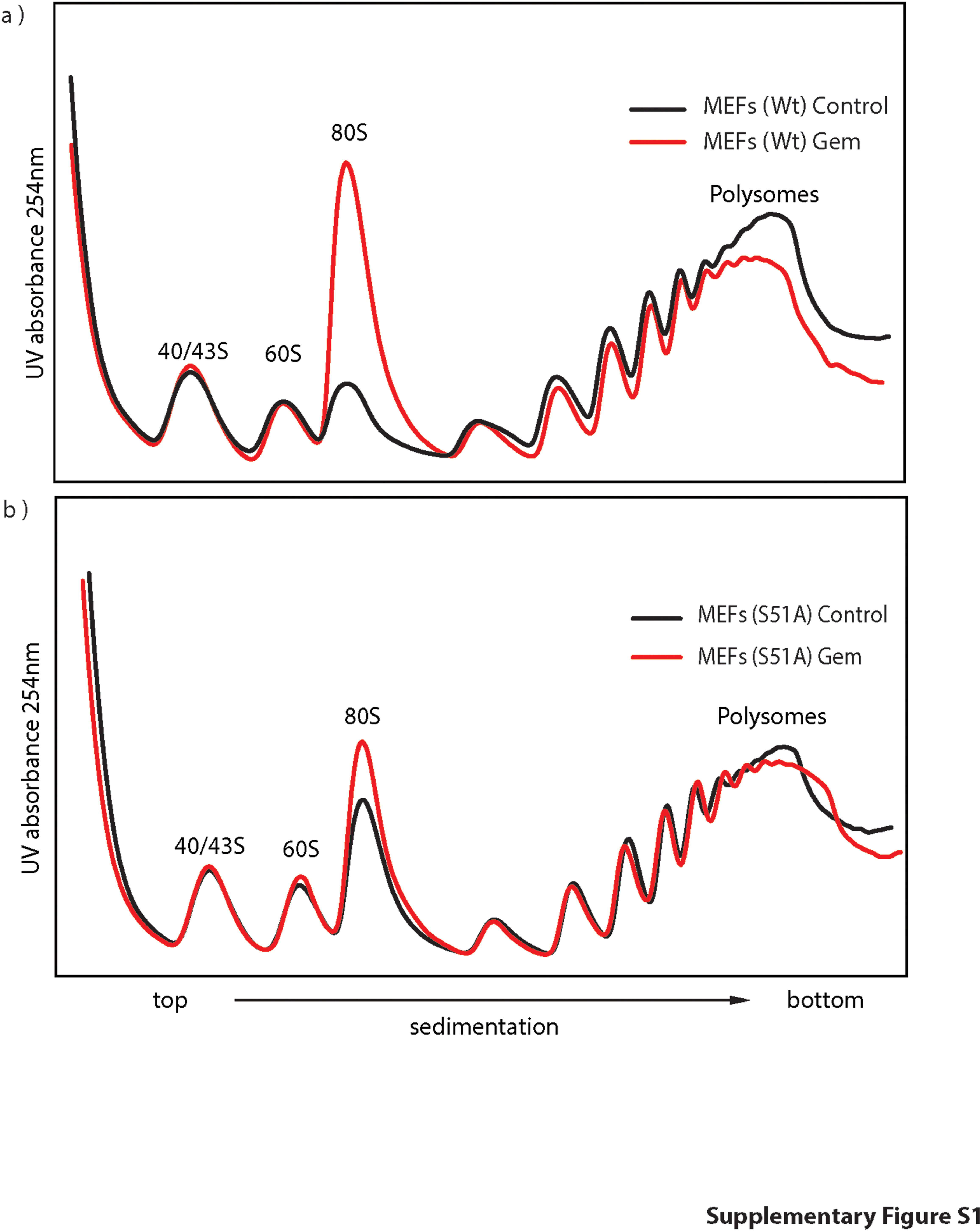

Supplement: Supplementary Figure S1 [file cddis2015264x2.tif]

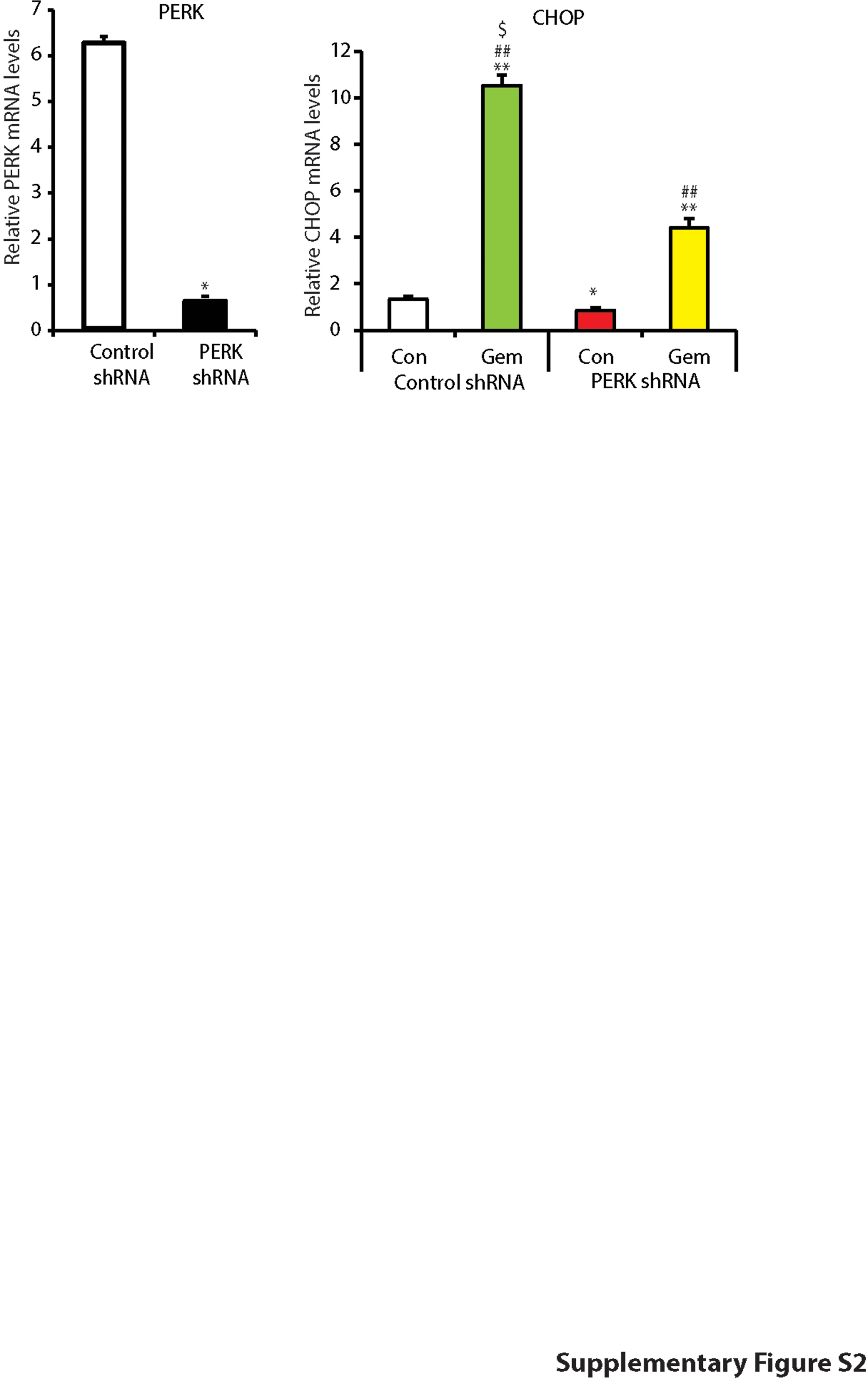

Supplement: Supplementary Figure S2 [file cddis2015264x3.tif]

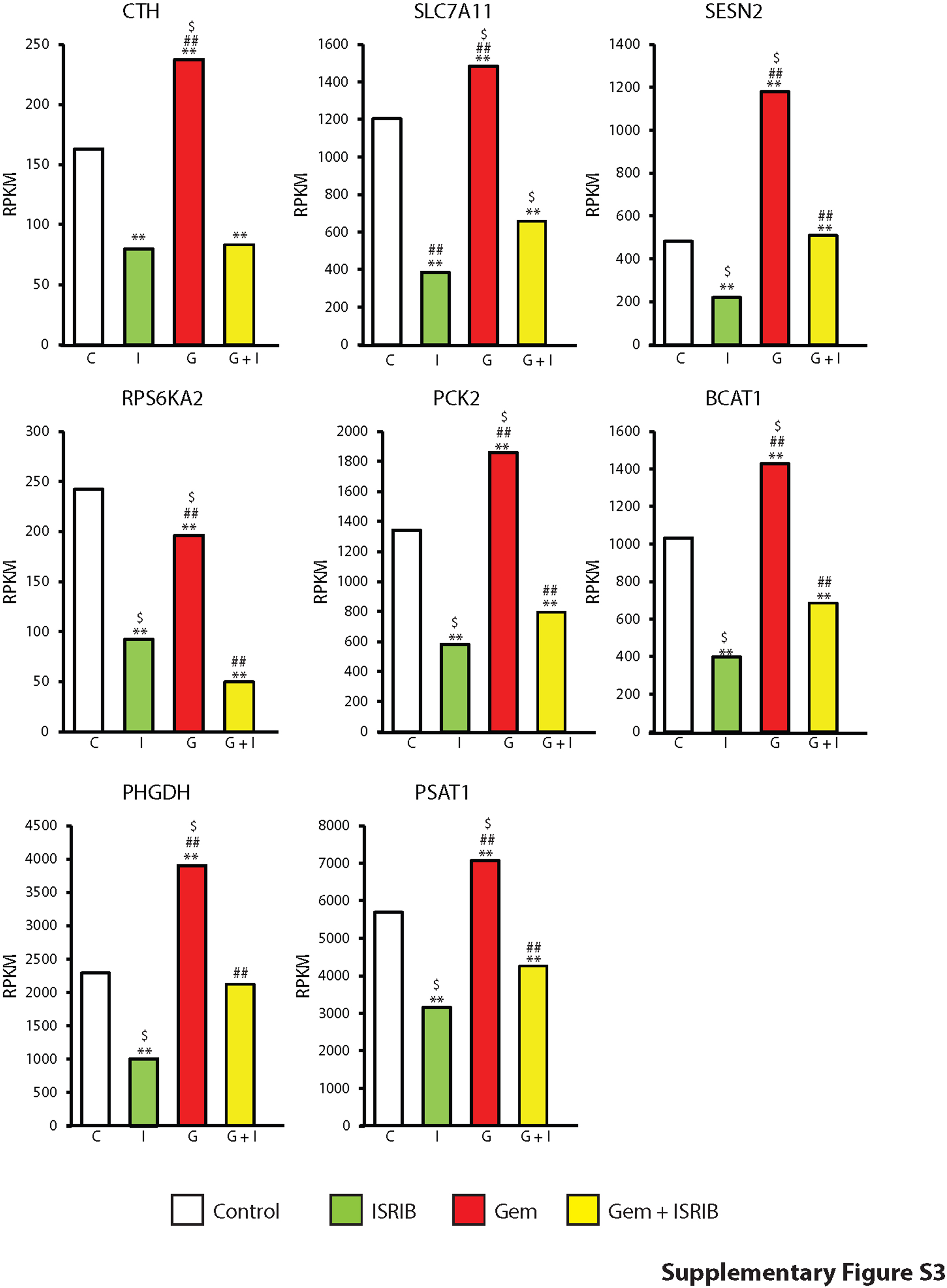

Supplement: Supplementary Figure S3 [file cddis2015264x4.tif]

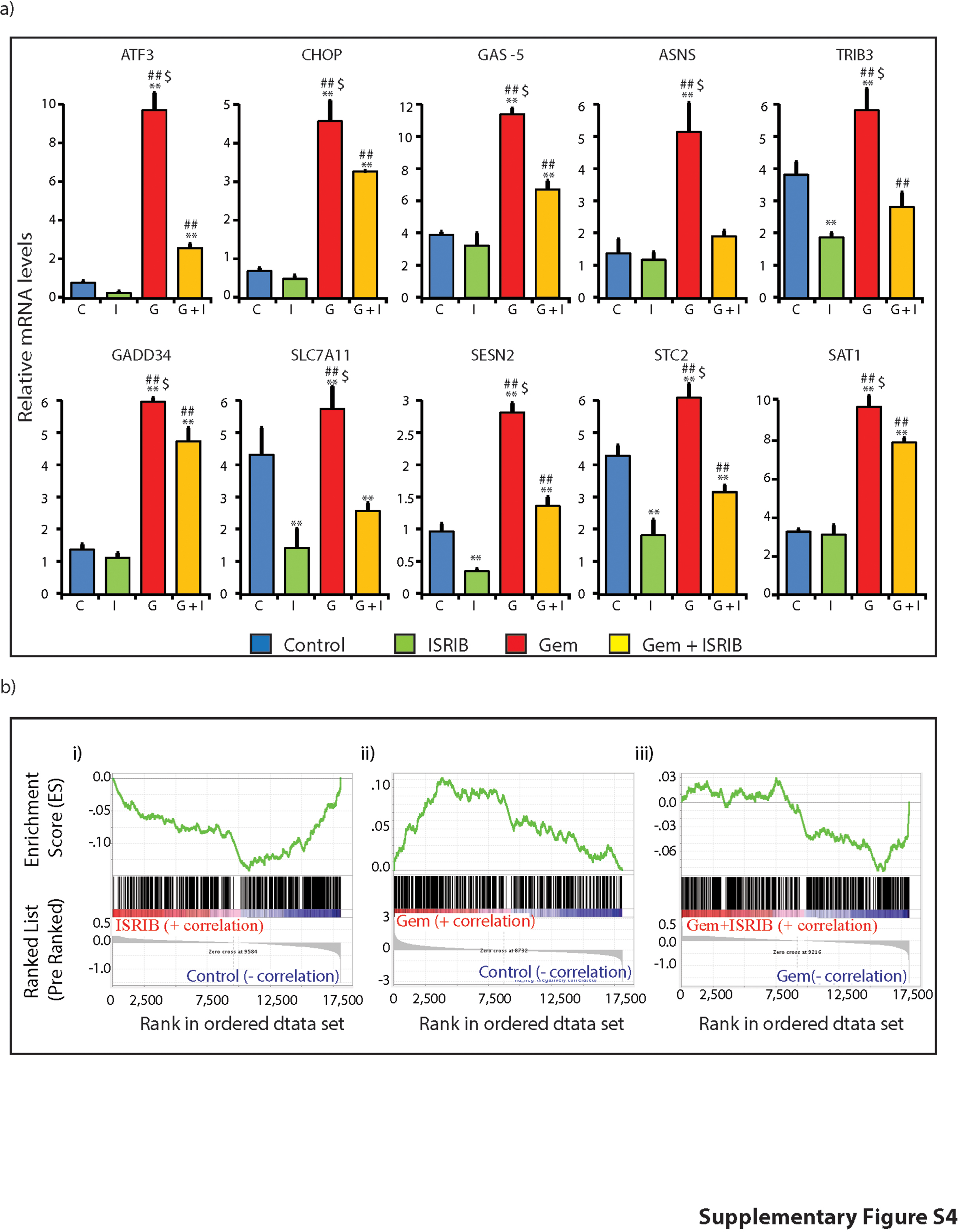

Supplement: Supplementary Figure S4 [file cddis2015264x5.tif]

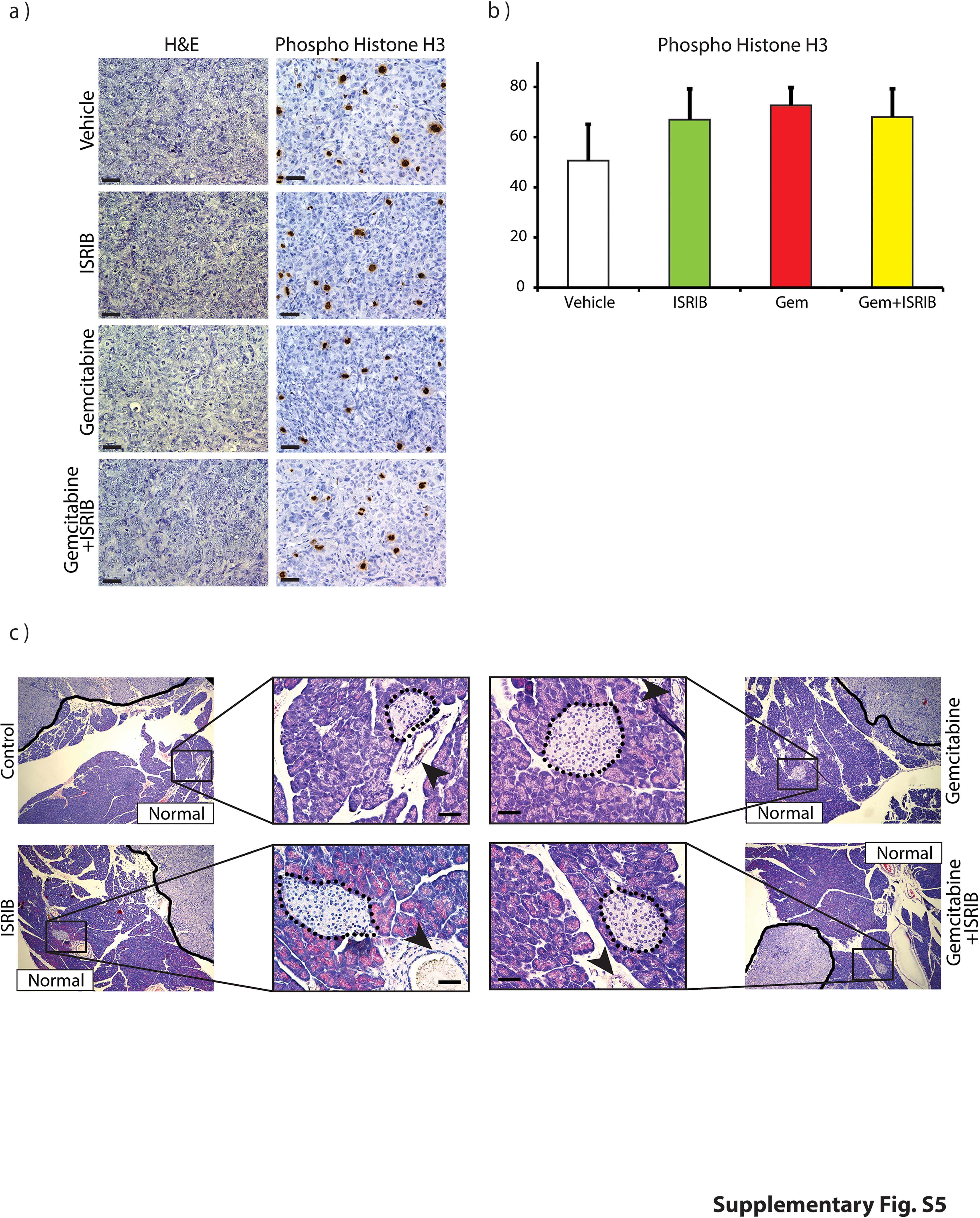

Supplement: Supplementary Figure S5 [file cddis2015264x6.tif]
